# A Systematic Approach to Capacity Strengthening of Laboratory Systems for Control of Neglected Tropical Diseases in Ghana, Kenya, Malawi and Sri Lanka

**DOI:** 10.1371/journal.pntd.0002736

**Published:** 2014-03-06

**Authors:** Janet Njelesani, Russell Dacombe, Tanith Palmer, Helen Smith, Benjamin Koudou, Moses Bockarie, Imelda Bates

**Affiliations:** 1 Capacity Research Unit, Department of International Public Health, Liverpool School of Tropical Medicine, Liverpool, United Kingdom; 2 Centre for Neglected Tropical Diseases, Department of Parasitology, Liverpool School of Tropical Medicine, Liverpool, United Kingdom; University of Maryland School of Medicine, United States of America

## Abstract

**Background:**

The lack of capacity in laboratory systems is a major barrier to achieving the aims of the London Declaration (2012) on neglected tropical diseases (NTDs). To counter this, capacity strengthening initiatives have been carried out in NTD laboratories worldwide. Many of these initiatives focus on individuals' skills or institutional processes and structures ignoring the crucial interactions between the laboratory and the wider national and international context. Furthermore, rigorous methods to assess these initiatives once they have been implemented are scarce. To address these gaps we developed a set of assessment and monitoring tools that can be used to determine the capacities required and achieved by laboratory systems at the individual, organizational, and national/international levels to support the control of NTDs.

**Methodology and principal findings:**

We developed a set of qualitative and quantitative assessment and monitoring tools based on published evidence on optimal laboratory capacity. We implemented the tools with laboratory managers in Ghana, Malawi, Kenya, and Sri Lanka. Using the tools enabled us to identify strengths and gaps in the laboratory systems from the following perspectives: laboratory quality benchmarked against ISO 15189 standards, the potential for the laboratories to provide support to national and regional NTD control programmes, and the laboratory's position within relevant national and international networks and collaborations.

**Conclusion:**

We have developed a set of mixed methods assessment and monitoring tools based on evidence derived from the components needed to strengthen the capacity of laboratory systems to control NTDs. Our tools help to systematically assess and monitor individual, organizational, and wider system level capacity of laboratory systems for NTD control and can be applied in different country contexts.

## Author Summary

Capacity strengthening activities such as technical training for staff, student research project supervision, and equipment provision are being carried out in laboratories worldwide as part of the global effort to control neglected tropical diseases (NTDs). However, these activities often focus on developing the skill sets of an individual and are not being thoroughly monitored and assessed. To address these gaps we developed a set of monitoring and assessment tools that can be used to determine the capacities required and achieved by laboratory systems to support the control of NTDs. The tools simultaneously focus on individuals (e.g., technicians, students, researchers), organisations (e.g., universities, research institutions, clinical facilities), national governments, and international agencies. Using the tools highlighted the strengths and limitations of each laboratory system in addition to the role of the laboratory regionally and internationally. We used the tools in Kenya, Ghana, Malawi and Sri Lanka, and concluded that our tools can be adapted and tailored to use in other countries and laboratories.

## Introduction

Effective prevention and treatment of neglected tropical diseases (NTDs) requires reliable and efficient laboratories for diagnosis and for supporting disease and entomological mapping surveys and yet laboratory systems are often weak in low and middle-income countries (LMICs) where the majority of this testing is carried out [1], [2]. Neglected tropical diseases consist of 17 microbiological diseases (see Table S1 for a list of the 17 Neglected Tropical Diseases as Classified by WHO) that affect the poorest people in the world. Current estimates suggest that over one billion people are infected with at least one NTD, and that these diseases cause approximately 534,000 deaths and 57 million disability adjusted life years (DALYs) each year [3]. In January 2012, as part of the London Declaration, a number of charities, pharmaceutical companies, and other businesses pledged to work together to improve the lives of people affected by NTDs and ultimately progress towards the elimination or control of 10 NTDs by 2020.

The lack of capacity in NTD laboratory systems in LMICs is a major barrier to monitoring and evaluation of interventions used for control and elimination of NTDs. The DFID funded Centre for Neglected Tropical Disease (CNTD) in the UK is monitoring the impact of mass drug administration (MDA) on the incidence of NTDs. The programme has found that lack of laboratory capacity in the CNTD supported countries is a critical bottleneck to implementing and monitoring community-based elimination interventions. To help the laboratories perform more effectively, the CNTD requested support from the Liverpool School of Tropical Medicine's (LSTM) Capacity Research Unit to design, monitor, and evaluate the capacity development of four laboratories in Ghana, Kenya, Malawi, and Sri Lanka.

Definitions of capacity development vary depending on the sector or particular programme focus, but a common definition is “ability of individuals, organisations or systems to perform appropriate functions effectively, efficiently and sustainably” [4]. Laboratory capacity strengthening is complex; it can require investment in specialised equipment, the support of all cadres of staff including laboratory scientists and researchers, as well as the leadership of the organisation in which the laboratory is housed, and sufficient time for training and embedding new processes, systems and equipment. Our aim was to develop a capacity strengthening programme which used a common approach to assessment and monitoring, but which could be tailored to take account of the different ways laboratories were financed, managed, and operated and their interactions with national programmes and regional collaborators. There are many capacity strengthening initiatives being carried out with laboratories in LMICs [5]; however, many of these initiatives focus on individuals' skills (e.g., technical skill of using microscope) [6] or institutional systems and processes (e.g., quality control office) [7] ignoring wider national and international structures (e.g., national and regional health systems) integral to establishing sustainable capacity.

In addition to the dearth of literature on organizational and national or international structures integral to capacity strengthening, rigorous approaches and methods to evaluate capacity strengthening initiatives are scarce [8]. Measuring the progress and impact of these capacity strengthening efforts is a priority for the international development community [9], but donors and scientists alike are struggling with how to do this well [5]. Evidence-based tools have been developed to help evaluate health research capacity strengthening [8] but in the area of laboratory capacity strengthening for NTD control and elimination specifically, no such tools exist.

The CNTD's goal in relation to laboratory capacity is to strengthen one laboratory in each of the four countries to support intervention activities that aimed to control and eliminate NTDs by 2020. To support this goal, our project aimed to describe and measure the capacities required by each laboratory at the individual (e.g., technicians, students, researchers), organizational (e.g., universities, research institutions, clinical facilities),, and national and international levels. To achieve CNTD's goal, our specific objectives were to a) use available evidence to describe the optimal capacities needed at each of the three levels for each laboratory if they were to achieve the goal, b) develop a set of assessment and monitoring collection tools that would enable us to assess what capacity gaps needed addressing if laboratories were to achieve optimal capacity and c) develop a capacity strengthening action plan to address the gaps and indicators that would enable us to monitor progress as capacity gaps were addressed.

## Methods

### Our approach to capacity strengthening evaluation

We used a validated framework and theory of change principles to guide the development of our capacity strengthening tools. The framework for designing and evaluating a health research capacity-building programme is based on four phases of capacity strengthening (see Table 1) - awareness, experiential, expansion, and consolidation [10]. Based on this framework an important first step in the awareness phase is to carefully review current capacity against a set of optimal standards and conduct a needs assessment to identify capacity gaps. We focused efforts on engaging all relevant stakeholders to determine the objectives of the capacity strengthening programme, identify capacity gaps and needs, and jointly develop a capacity development action plan. Our approach enabled stakeholders to be actively involved in the assessment and monitoring process. To carry out these activities we recognized that we would require specific assessment and monitoring collection tools and would need to consult various data sources within each laboratory system.

**Table 1:** Framework for designing and evaluating a health research capacity-building programme.

Awareness	Efforts made to engage all relevant stakeholders at organisation and policy level as well as individuals involved in implementing capacity strengthening (CS) cycle; emphasis on local ownership with defined role for external input
Experiential	Plans for CS, with timelines, developed in collaboration with all stakeholders with external input; local change agents identified; start small, test and intensively monitor different models; plans implemented in a continuous learning cycle
Expansion	Identify scalable models and easy-to-measure indicators for long-term monitoring; New capacity becomes embedded in existing structures; build on strengths and what works; efforts to influence policy and identify sustainable funds
Consolidation	Capacity development becomes routine, independent funds secured, minimal external input, autonomy to be flexible and solve problems.

We also draw on theory-based evaluation methods, particularly theory of change evaluation, to develop our approach to laboratory capacity strengthening. We define theory of change as “An on-going process of reflection to explore change and how it happens – and what that means for the part organisations play in a particular context, sector and/or group of people” [11]. Using a theory of change approach involves specifying an explicit theory of how and why a capacity strengthening intervention might cause an effect, and this is used to guide the evaluation [12]. Guided by this, our theory was that strengthening laboratories for NTD control is a complex and non-linear process involving wider systems and actors beyond the institution; we also assumed strengthening capacity in the laboratories would involve strengthening partnerships, organisational development, empowering people, and open communication. We purposely choose to incorporate theory of change in our work in order to determine indicators that could help us explore the relationship between the programme inputs, activities, and outcomes.

### Development of assessment and monitoring tools

Prior to our research, no tools existed for specifically examining the capacities required by laboratory systems at the individual, organizational, and national and international levels to support the control of NTDs, or for capturing information from various data sources within laboratory systems. Therefore we developed our own tools based on evidence concerning the components (i.e., people, skills, systems, resources) needed to strengthen the capacity of laboratory systems. We used a three-stage approach to develop the assessment and monitoring tools. First we searched published evidence concerning laboratory capacity strengthening at the individual, organisation, and national and international system level. We searched the electronic databases of PubMed and Google Scholar, using the keywords “laboratory”, “NTD” and “capacity strengthening”. We also consulted books and published reports concerning capacity strengthening initiatives conducted with medical laboratories. From this information we were able to generate a list of all the components that were necessary for an optimal laboratory system in the domain of NTDs and used this to inform the design of our tools. Specifically, the following documents guided the development of our assessment and monitoring tools; the Global Laboratory Initiative Stepwise Process towards TB Laboratory Accreditation [13] and adapted for NTD laboratories, the EFQM excellence model [14], the SIDA evaluation model of HEPNet [15] and the UNDP Measuring Capacity document [16]. Using all the components in the list of optimal capacities we developed a questionnaire for laboratory managers, a semi-structured interview guide for use with laboratory stakeholders, a capacity gap checklist for use with the laboratory manager and laboratory staff, and a checklist for ISO 15189 to be used for on-site observations (see Table 2). Our intention was to use these tools during a site visit to collect data that would allow us, in collaboration with local stakeholders (e.g., laboratory technicians, laboratory managers, NTD scientists, directors of institutions, Ministry of Health representatives, etc.), to identify capacity gaps and to create a comprehensive capacity development action plan to address the gaps.

**Table 2:** Assessment and monitoring tools.

Tools	Purpose	Target group	Content areas
**Questionnaire**	To understand existing laboratory capacity and capacity gaps, and access background information about the laboratory	Laboratory managers	Organizational structure, strategic planning, local, national & international stakeholders, national and regional collaborations and MOU's, funding, national and regional NTD laboratory functions, current capacity and gaps
**Semi-structured interview**	To determine existing laboratory capacity, identify capacity gaps, and challenges to strengthening capacity	Individuals an interest in changes and developments in the capacity of the laboratory including: the NTD programme manager, representatives of donor organisations, heads of other laboratories in the national network, representatives of academic or research institutions, and technical advisors in NTDs in the country	Laboratory organization and strategic planning, organizational learning, external partnerships and collaborations, laboratory research activities, the regional laboratory network
**Capacity gap checklist**	To determine existing laboratory capacity, identify capacity gaps and challenges to strengthening capacity	Staff employed directly or indirectly by the laboratory, including: laboratory manager, laboratory scientists, research staff, technical and support staff, students, and HR/financial staff	Laboratory strategy and communications, opportunities for organizational learning, external interactions, financial resource management, people and equity, research activity, regional networking
**ISO checklist**	To gauge readiness for ISO 15189 accreditation	Laboratory scientists	Safety, equipment, infrastructure, supply chain, specimen management, quality monitoring, personnel management, requesting and reporting, data and document management, client communication, and organization & finance

### The assessment and monitoring tools

#### Questionnaire

The questionnaire aims to introduce the concept of our capacity strengthening programme and to capture the immediate challenges faced by the laboratories in meeting its goal. The questionnaire begins the process of assessing the needs of each laboratory with questions pertaining to laboratory organization, position within the national laboratory network, and relationship to the wider national and regional health system. The questionnaire is designed to be completed electronically by the director or manager of each of the four NTD laboratories 2–3 weeks in advance of the site visit to undertake the full needs assessment. The rationale for sending out the questionnaire beforehand is for the laboratory manager to begin thinking about current capacity and potential gaps, and for us, as independent partners in capacity strengthening design and evaluation, to access some background information about the laboratory. The questionnaire is intended to be completed fairly rapidly (less than 30 minutes), returned by e-mail, and to be followed up with further communication as needed to clarify information and data sources and any other issues from both sides.

#### Interview guide

The purpose of the interview is to engage the laboratory's main stakeholders in face-to-face discussions about existing capacity in the laboratory, and through a series of prompting questions, identify priorities and challenges to strengthening capacity. The main topics included in the interview guide are organisation and strategic planning of the laboratory, creating opportunities for the laboratory as an organisation to learn, external partnerships or collaborations, national and regional role, and research activities undertaken. These topics were derived from literature concerning what was considered to be the optimal for the goal of these laboratories.

The interview guide is designed for use with a wide group of stakeholders who have an interest in, or who are affected by, changes and developments in the capacity of the laboratory. Across the four countries we worked with in this project, we interviewed range of stakeholders including: NTD programme managers, representatives of donor organisations, heads of other laboratories in the national network, representatives of academic or research institutions, Government representatives (particularly Ministry of Health staff), and other technical advisors for NTDs in the country.

#### Capacity gap checklist

The capacity gap checklist is designed for different cadres of staff employed at the laboratory (e.g., laboratory scientists, research staff, technical and support staff, students, and HR/financial staff) to complete in order to obtain multiple views on existing capacity, gaps, and strengths of the system. The criteria in the checklist represent the optimal capacity needed to achieve an effective and sustainable laboratory with capability to meet NTD programme needs. Based on common criteria we identified in the literature, the checklist included the following key areas: laboratory strategy and communications, opportunities for organizational learning, external interactions, financial resource management, people and equity, research activity, and regional networking. The checklist also has a column for individuals to record their assessment of current capacity against optimal capacity criteria using a score of 1–4 (1 = no agreement; 4 = maximal agreement) and a column to record any explanation of the assigned assessment score. The checklist can also be used to list any supporting documentation relevant to each criterion and space to record sight of such documents, date of publication, and review dates. Following completion of the capacity gap analysis checklist by each individual, the data gathered from the checklist is analyzed to highlight the strengths, gaps, and discrepancies between laboratory members. Discrepancies are resolved through discussions with each subsequent laboratory stakeholder until consensus is reached.

#### ISO 15189 checklist

As part of the needs assessment with each laboratory, we wanted to gauge how well the laboratories were equipped, set up, and managed, and to do this we designed a checklist based on ISO15189 standards. As the study was carried out with NTD research laboratories, the ISO checklist did not include laboratory functioning domains outside the scope of a research laboratory such as participation in surveillance and response activities. The ISO checklist is completed with the laboratory manager and safety and quality officers to identify specific gaps to overcome in the short term, and what is required in the longer term to achieve ISO 15189 accreditation. This checklist is derived from the WHO laboratory quality management system training toolkit [17] and the Global Laboratory Initiative (GLI) Stepwise Process towards TB Laboratory Accreditation [13]. The GLI process is specifically targeted at tuberculosis reference laboratories so some of the specific content required changing to be relevant to NTDs. ISO accreditation is considered the gold standard for clinical laboratory accreditation internationally. Our checklist is designed as a simple tick box exercise and includes the topics of safety, equipment, infrastructure, supply chain, specimen management, quality monitoring, personnel management, requesting and reporting, data and document management, client communication, and organisation and finance.

### Data analysis

We analyse the data generated from all the tools using content and thematic analysis. Specifically, we use an analytic framework to help guide thematic data analysis of the interview and focus group data. The analytic framework consists of a range of apriori codes that help to organize the data generated and includes codes pertaining to quality assurance, institutional collaboration, funding, NTD coverage or focus, research capacity, and organizational resources. Data from the checklists and questionnaire are analysed using content analysis.

### Developing capacity development action plans with each laboratory

We use the findings of the capacity gap analysis to jointly develop with laboratory managers their own unique five-year capacity development strategy to improve their capacity to conduct research and analysis to support NTD control. Gaps in capacity that need to be filled to achieve the strategy are agreed upon during a consensus meeting with invited stakeholders. Priority gaps that require action in the first year are proposed by stakeholders and amalgamated into a one year capacity development action plan with measurable indicators and targets to drive capacity strengthening. The plans are then finalised through Skype and email discussions (e.g., details concerning completion dates) after the completion of each of the visits. These capacity development action plans can also be used to mobilize donor funding as they highlight and provide justification for the priority areas where funding needs to be invested.

### Implementation of the tools

Following development of the tools, we implemented them in four of the CNTD/LF programme (2012-16) funded laboratories, including Ghana, Kenya, Malawi, and Sri Lanka. The laboratories in each country were initially selected by CNTD to be a part of their MDA programme because it had been identified that a lack of capacity globally in laboratory systems was a major bottleneck in the monitoring of MDA. Of all of the laboratories in the MDA programme, the laboratories in Ghana, Kenya, Malawi, and Sri Lanka were chosen to be a part of the pilot study because each were seen to be potential regional leaders in the control of NTD and had a potential ability to support NTD laboratories in other countries. See Table 3 for a description of each laboratory involved in the study. Implementation of the tools occurred throughout 2012 during a 5–10 day visit at each institution, with two complementary members (e.g., laboratory specialist, social scientist) of the Capacity Research Unit leading each visit.

**Table 3:** Description of laboratories included in our project.

	CNTD partner countries included in our capacity strengthening programme			
	Ghana	Malawi	Kenya	Sri Lanka
**Human resources**	5 part-time staff: 1 secretary, 4 scientists	6 full-time staff: 1 director, 1 laboratory technician, 1 senior scientist, 3 research assistants	7 full-time staff: 5 laboratory technologists, 1 research assistant, 1 principal research scientist, support from director and lab-in-charge	34 full-time staff: 1 director, 1 laboratory supervisor, 4 public health laboratory technicians, medical officers
**Governance**	Under Ministry of Education	Under the Malawi College of Medicine	Under the Kenyan Medical Research Institute	Under the Ministry of Health
**Priority functions**	Research, lymphatic filariasis training	Research	Research, international training courses	Parasitic and vector surveillance, routine monitoring, deformity care (e.g., patent education), staff training, and research studies
**Disease programme**	Lymphatic filariasis	Lymphatic filariasis, malaria	Lymphatic filariasis	Lymphatic filariasis
**Level of functioning**	Provides support, mostly training, to national lymphatic filariasis programmes across the continent.	Extended remit from operational research support for malaria to all communicable diseases including lymphatic filariasis.	Laboratory is operational.	Responsible for all lymphatic filariasis activities including control, monitoring and surveillance across three provinces.
**Networks**	Collaborates with national NTD programme that coordinates all NTD/LF activities, including control, monitoring and surveillance, across Ghana.	Collaborates with national lymphatic filariasis programme that is responsible for all lymphatic filariasis activities including control, monitoring and surveillance across all lymphatic filariasis endemic districts in Malawi.	Collaborates with National NTD programme in the Ministry of Health.	Collaborates with Medical Research Institute, whose mandate is to perform research, surveillance, quality control, teaching and training.

A total of 62 semi-structured interviews were conducted, 17 in Malawi, 11 in Ghana, 16 in Kenya, and 18 in Sri Lanka. We interviewed stakeholders from a range of institutions and levels including laboratory scientists, laboratory directors, research staff, WHO staff, ministry representatives, students, human resource and financial staff, donors, and senior academics. For example, key NTD stakeholders in Kenya were drawn from the Eastern and Southern Africa Centre of International Parasite Control NTD laboratory located in the Kenyan Medical Research Institute and the National NTD programme through the office of the Department of Disease Prevention and Control in the Ministry of Health. In addition to the semi-structured interviews, in each country one pre-visit questionnaire and ISO checklist were completed, 2–4 capacity gap checklists were completed, and one focus group was held.

### Revising the tools

We revised the tools after their implementation in each country by conducting a retrospective analysis of how the tools contributed or not to the awareness phase in the framework for designing and evaluating a health research capacity-building programme that guided the design of our capacity strengthening tools. The analysis was developed through collaborative and candid dialogue by the research partners, using the framework as the basis for deliberation. These analysis meetings with the entire research team reviewing the findings were an important step in establishing rigour in the refinement of the tools. Throughout the analysis, questions were asked such as; “Were all relevant stakeholders at organisation and policy level as well as individuals involved in implementing capacity strengthening cycle engaged?” and “Was there an emphasis on local ownership with defined role for external input?” Results of the retrospective analysis shed light on factors such as how some stakeholders were not participating in the capacity assessment possibly as a result of the work being carried out in a context where being critical could be considered inappropriate, particularly for a junior member of staff.

To address this particular issue, we adapted the methods to include focus group discussions specifically for laboratory staff, where laboratory managers did not participate. These refinements enabled us to gain an increasingly greater depth and breadth of information from laboratory staff. The retrospective analysis also illuminated that the laboratories held varying capacity strengths and gaps and the tools needed to be able to be tailored accordingly. For example, following the work in Malawi, modifications of the tools included re-designing the ISO checklist to enable laboratory staff to bypass sections of questions that were not relevant to their laboratory's stage of development. By analyzing the implementation of the tools in succession in different countries we had time to use systematically lessons we had learnt to revise the tools between each evaluation.

### Ethics

We obtained ethics approval for the capacity strengthening component of the work from the LSTM Research Ethics Committee. The wider DFID funded CNTD programme has ethics approval for all monitoring and evaluation activities scheduled to be implemented in the country laboratories.

## Results

### Existing strengths and gaps in laboratory capacity to support NTD research and monitoring

Using the rich information collected with the tools we were able to identify strengths and gaps in NTD laboratories' systems capacity (see Table 4). The identified strengths and gaps varied amongst the countries; however, inter-laboratory comparison revealed some similarities. For example, all laboratory systems mentioned that NTDs being recognized as a national priority was a specific strength, which resulted in greater availability of national funding and human resource support for laboratories. The following quote from a stakeholder in Kenya illustrates this finding, *“A national multi-year strategic plan for control of NTD was published in 2011”*. Furthermore, in all countries the laboratories had strong links to policymakers and existing national and regional collaborations.

**Table 4:** Comparison of existing strengths in laboratories included in our project.

	CNTD partner countries included in our capacity strengthening programme			
Existing laboratory capacity strengths	Ghana	Malawi	Kenya	Sri Lanka
**People and management**	Skills and abilities matched to needs of laboratory.	Young, expanding research and technical laboratory team to support LF/NTD work.	Flexible laboratory scientist capacity.	34 full-time staff with four experienced laboratory scientists.
**Research support**	Research office responsible for overseeing all research.	Code of practice for research and institutional support for grant writing and funding.	Code of practice for research, grantmanship office, and ethics review committee.	All research goes through Ministry of Health ethics committees.
**External interactions**	Works with a range of partners, across all sectors within the local and international community.	Offers of support from other local laboratories to develop quality and safety systems.	Local expertise and support available to develop the NTD laboratory. East African Laboratory to support refurbishment of laboratory. Training of staff from external organizations.	International Filariasis Research group supported research and surveillance.
**National and regional collaborations**	Collaborates with national NTD programme.	Close links with National LF programme.	Strong partnerships universities exist.	Links with Medical Research Institute.
**Policy-maker engagement**	Strong links with policy makers and is housed within Ministry of Education.	Strong national policy influence in malaria, but not for NTDs.	Close links with national NTD programme.	Based within the Public Health Complex in the Ministry of Health.

In regards to capacity gaps, one common gap was the lack of funding for NTD research, as allocating funding for research was seen as less of a priority than operations and management when health sector funding decisions were being made. Also common to all of the laboratories was a lack of quality assurance documentation and safety systems, a lack of formalized agreements with national NTD programmes, and reliance on external funds. There also was a specific disease focus in each laboratory, without consideration of the broader NTD focus, creating a need for each laboratory to consider how they move beyond their specific focus on malaria or lymphatic filariasis etc. to NTDs as a whole. Finally, there was a lack of research and biostatistics capacity in all of the laboratories, partially due to the fact that research training courses were not accessible to all staff.

### Laboratory readiness for ISO accreditation

Activities were identified for each country to undertake to work towards achieving ISO 15189. As with the strengths and gaps, the identified activities varied amongst the countries; however, inter-laboratory comparison revealed some similarities. The checklist revealed that none of the countries had written safety systems in place (e.g., procedures to follow in event of a biohazardous incident that are essential to achieve quality assurance). Therefore, similar activities that needed to be undertaken in each country included the drafting of full standard operating procedures for all experimental processes, safety, and equipment in the laboratory. Additional gaps in relation to ISO standards included the need to appoint and assign a safety officer and to have job descriptions available for all staff.

### Laboratories' potential to provide support to national and regional NTD control programmes

The tools generated information about how the NTD laboratories could support national NTD programmes in the region with achieving their aims. The NTD laboratories were found to provide timely and helpful input on country specific issues for topics related to NTDs such as sample diagnostics, vector analysis, and the efficacy of control programmes. For example, in Kenya the tools helped identify the potential for the laboratory to provide support to regional LF control programmes in Tanzania, Zimbabwe, Botswana, and Zambia. Additional potential activities that were identified through our process include confirmation of NTD elimination through implementation of monitoring and evaluation activities, quality control, processing of samples collected through operational research carried out in hotspot areas where transmission of NTD is persisting even after several mass interventions, and support other operational research activities aimed to support implementation. Furthermore, in each country the laboratories were found to provide robust scientific data to support national and regional NTD control programmes, enabling policy makers to make informed decisions that contributed to control and elimination of NTDs in their country and region.

### Laboratory's position within national and international networks and collaborations

Information about each NTD laboratory's position within national and international networks and collaborations was generated from the set of tools. Findings indicate that the level of technical expertise and experience within the laboratory system enhanced a laboratory's position within their networks as with this expertise the laboratory was seen to be a preferential collaborator. Technical expertise was perceived by stakeholders to be more essential to a laboratory's position within networks than other factors such as geographic proximity. For example, the laboratory scientists in Ghana are highly skilled in using real-time polymerase chain reaction (RT-PCR). Given their expertise the Ghanaian scientists were identified as being able to provide training to other laboratories within the CNTD network.

## Discussion

We have described our systematic process for developing evidence-based, practical ways of assessing and monitoring the capacity of laboratories in LMICs to contribute to NTD control and elimination. The set of tools we have developed help to systematically evaluate individual, organizational and system level capacity of laboratory systems for NTD control. Using the tools enabled the stakeholders and researchers to jointly develop a capacity development action plan that aimed to control or eliminate NTDs in their region. We had multi-level stakeholders involved, including laboratory staff, administrators, international organization representatives, academics, and policy makers. This creation of partnerships with a range of decision makers is known to be an effective strategy to strengthen capacity [18], [19]. The literature in the field highlights that assessment and monitoring is more often driven by those outside of the country such as donors who are often concerned with conducting fiscal assessments [20].

While the importance of individual and institutional capacity has been raised in the literature [21], this study is novel as it explores capacity within laboratory systems at the national and regional levels. The tools enabled us to explore outcomes beyond the individual level such as understanding the strengths and gaps at the organizational level (e.g., relationship between NTD laboratory and College of Medicine in Malawi). Through exploration of capacity at the organization level, it was revealed that there is a need for each laboratory to consider how they can move beyond their one specific NTD focus. This consideration of moving to a broader focus could even include discussion of the integration of NTDs into the control of the big three i.e., tuberculosis (TB), malaria, and HIV. Potential synergies between the Global Fund diseases of malaria, HIV and TB were identified by the NTD community many years ago [22]. They are all diseases of the poor and co-endemic with at least one NTD across the distribution of the WHO focus NTDs. Initially, the focus was on optimizing delivery strategies and building on common features in the supply chain management system to scale up intervention coverage in a highly cost effective way. As NTD laboratories embark on scaling up through inter-sectoral approaches, they could also capitalise on the growing support for reference laboratories, through the Global Fund for AIDS, Tuberculosis and Malaria (GFATM). NTD diagnostics could be included in the activities of these national reference laboratories. Diagnosis for the Global Fund diseases are commonly achieved using rapid diagnostic procedures based on small quantities of finger-prick blood samples that also can be used to test for many NTDs, as can DNA extracted from blood.

Using the tools also gives credence to the idea that capacity resides at different levels, including individual, institutional, national and regional but is best addressed institutionally. Addressing capacity strengthening initiatives at the institutional level is congruent with principles within theory of change evaluation which emphasise that organisations and individuals within them have a key role to play in moving from one state of capacity to another, while also acknowledging the contribution and influence of other actors outside the organisation's control [11]. Taking this systemic view, capacity strengthening can be conceptualised as a process of change within a complex system of unpredictable interactions and inter-relationships between elements and individuals. A small change in one aspect or relationship can have a significant impact on capacity, and the key to success is in observing and capturing these changes which often happen in a non-linear way. Although we only have implemented the tools in four countries thus far, the commonalities across cases suggest that our tools are appropriate for a range of contexts. We found value in transferring the tools from thee different African contexts to a South East Asian context, as the tools were found to be flexible enough to be adapted to the different country context and enabled us to collect relevant data and monitor progress in capacity strengthening. This flexibility in the tools, allowing for adaptation to different contexts, has been shown to enhance capacity strengthening initiatives [10]. We believe therefore that the tools could be used in laboratory systems beyond the scope of NTDs and would encourage further research to examine this.

This study contributed to the literature about how to assess and monitor capacity strengthening in practice. Through using the tools we learnt more about the process of capacity strengthening including the recognition that personal relationships are key to capacity strengthening initiatives. Assessing and monitoring indicators such as relationships amongst stakeholders (e.g., laboratory director and national program) is far less tangible than indicators used in the bulk of capacity strengthening research (e.g., number of people trained) [23]. This finding leads to the recognition of the value of using mixed research methods to measure changes in capacity, rather than the traditional approach of predominantly quantitative measures [24] in order to obtain an in-depth understanding of complex constructs and inter-relationships that operate in health systems.

### Conclusion

Our novel set of assessment and monitoring tools provide a practical and field-tested approach for assessing laboratory capacity strengthening initiatives. We have implemented the tools for laboratory system strengthening in NTD laboratory systems in three countries in Africa and one country in South East Asia, but they could be adapted for use in other geographical and laboratory contexts.

## Supporting Information

Table S1 **The 17 Neglected Tropical Diseases as classified by WHO.**
(DOCX)
