# Supplementary material for: A Systematic Approach to Capacity Strengthening of Laboratory Systems for Control of Neglected Tropical Diseases in Ghana, Kenya, Malawi and Sri Lanka
Source: PLoS Negl Trop Dis. 2014 Mar 6;8(3):e2736. doi: 10.1371/journal.pntd.0002736 (PMC3945753; doi:10.1371/journal.pntd.0002736)
Supplement: Table S1 — The 17 Neglected Tropical Diseases as classified by WHO. (DOCX) [file pntd.0002736.s001.docx]

**Table S1: The 17 Neglected Tropical Diseases as Classified by WHO**

Buruli Ulcer (Mycobacterium ulcerans infection)

Chagas disease

Dengue/Severe dengue

Dracunculiasis (guinea-worm disease)

Echinococcosis

Foodborne trematodiases

Human African trypanosomiasis (Sleeping sickness)

Leishmaniasis

Leprosy

Lymphatic filariasis

Onchocerciasis (River blindness)

Rabies

Schistosomiasis

Soil transmitted helminthiases

Taeniasis/Cysticercosis

Trachoma

Yaws (Endemic treponematoses)

WHO (2013) ‘Neglected Tropical Diseases’ [Online] Available at: <http://www.who.int/neglected_diseases/diseases/en/> Accessed 23/10/2013
